# Publisher Correction: Carrier lifetime enhancement in halide perovskite via remote epitaxy

**DOI:** 10.1038/s41467-019-12571-1

**Published:** 2019-10-16

**Authors:** Jie Jiang, Xin Sun, Xinchun Chen, Baiwei Wang, Zhizhong Chen, Yang Hu, Yuwei Guo, Lifu Zhang, Yuan Ma, Lei Gao, Fengshan Zheng, Lei Jin, Min Chen, Zhiwei Ma, Yuanyuan Zhou, Nitin P. Padture, Kory Beach, Humberto Terrones, Yunfeng Shi, Daniel Gall, Toh-Ming Lu, Esther Wertz, Jing Feng, Jian Shi

**Affiliations:** 1 Department of Materials Science and Engineering, Kunming University of Science and Technology, Kunming, Yunnan 650093 China; 2 Department of Materials Science and Engineering, Rensselaer Polytechnic Institute, Troy, 12180 USA; 3 Department of Physics, Applied Physics and Astronomy, Rensselaer Polytechnic Institute, Troy, NY 12180 USA; 4 State Key Laboratory of Tribology, Tsinghua University, Beijing, 100084 China; 5 Beijing Advanced Innovation Center for Materials Genome Engineering, Institute for Advanced Materials and Technology, University of Science and Technology Beijing, Beijing, 100083 China; 6 Ernst Ruska-Centre for Microscopy and Spectroscopy with Electrons and Peter Grünberg Institute, Forschungszentrum Jülich, Jülich, Germany; 7 School of Engineering, Brown University, Providence, RI 02912 USA; 8 Center for Materials, Devices, and Integrated Systems, Rensselaer Polytechnic Institute, Troy, NY 12180 USA

**Keywords:** Materials science, Materials for energy and catalysis, Solar cells

Correction to: *Nature Communications* 10.1038/s41467-019-12056-1, published online 12 September 2019.

The original version of this Article contained an error in the author affiliations. The fifth affiliation incorrectly read ‘Beijing Advanced Innovation Center for Materials Genome Engineering, Institute for Advanced Materials and Technology, University of Science and Technology, Beijing 100083, China.’ The correct version reads ‘Beijing Advanced Innovation Center for Materials Genome Engineering, Institute for Advanced Materials and Technology, University of Science and Technology Beijing, Beijing 100083, China.’ This has now been corrected in both the PDF and HTML versions of the Article.

