# Supplementary material for: Carrier lifetime enhancement in halide perovskite via remote epitaxy
Source: Nat Commun. 2019 Sep 12;10:4145. doi: 10.1038/s41467-019-12056-1 (PMC6742762; doi:10.1038/s41467-019-12056-1)
Supplement: Supplementary file 1 — Supplementary Information [file 41467_2019_12056_MOESM1_ESM.pdf]

Supplementary Information

for

Carrier Lifetime Enhancement in Halide Perovskite via Remote Epitaxy

Jiang et al.

Supplementary Figures

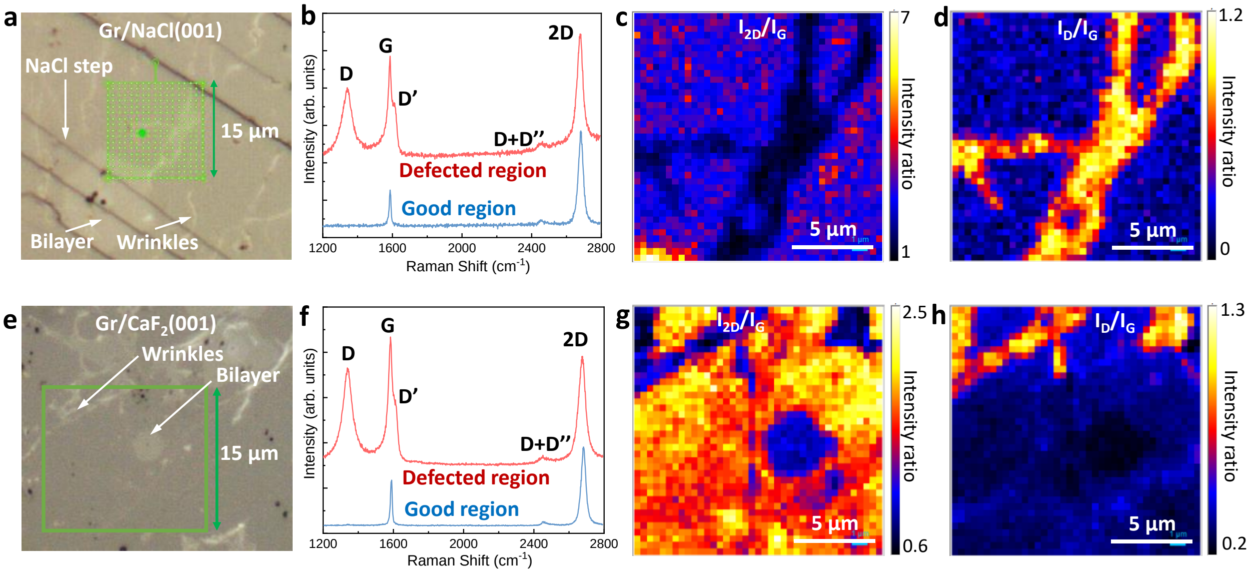

Supplementary Figure 1 Raman measurements on transferred graphene. Optical images of transferred graphene on NaCl (**a**) and CaF_2 (**e**). Raman spectra of both defected and good regions of graphene on NaCl (**b**) and CaF_2 (**f**). Raman mapping of I_{2D}/I_G and I_D/I_G of graphene on NaCl(001) (**c** and **d**) and CaF_2(001) (**g** and **h**), respectively. The mapping area of 15×15 μm^2 is indicated in green square in **a** and **e**.

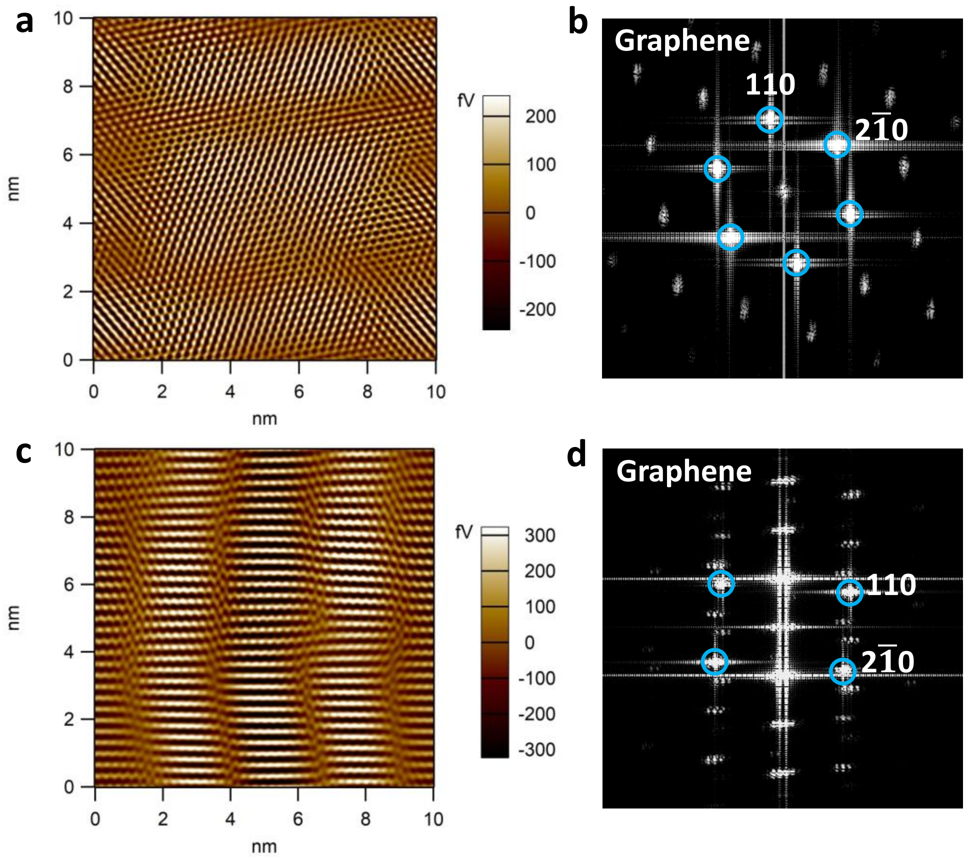

Supplementary Figure 2 HRAFM of graphene on NaCl(001) (**a**) and CaF_2(001) (**c**) and their FFTs (**b** and **d**, respectively).

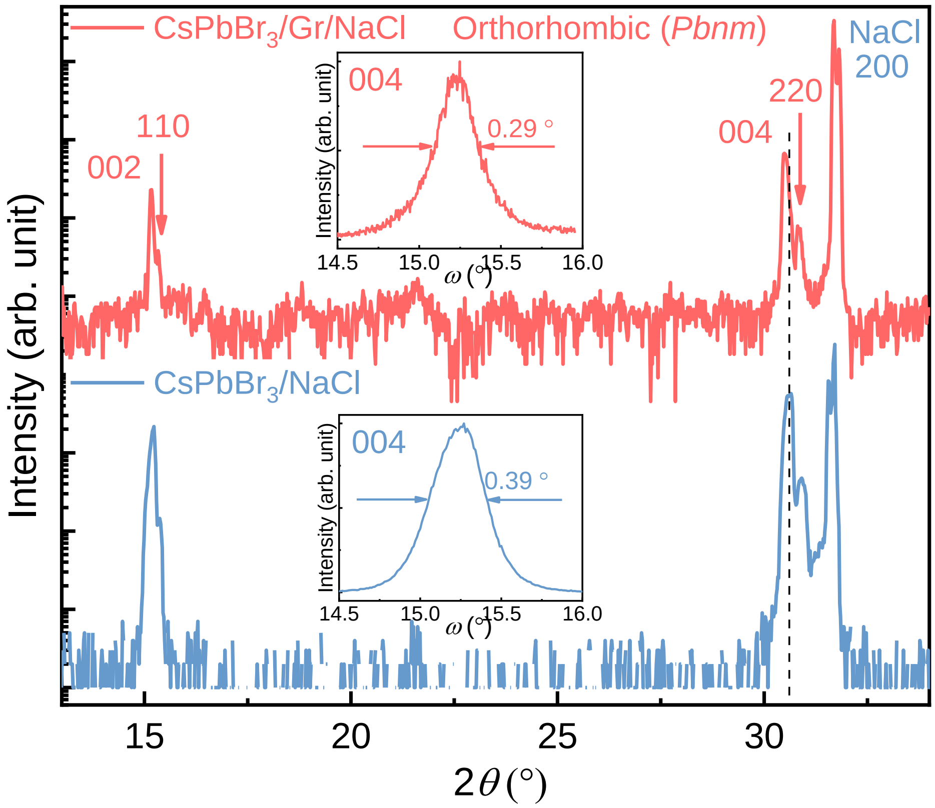

Supplementary Figure 3 XRD θ-2θ scans for remote epitaxial $CsPbBr_3$ flakes on Gr/NaCl (upside in red color) and ionic epitaxial $CsPbBr_3$ film on NaCl (downside in blue color), the corresponding rocking curve are in the insets.

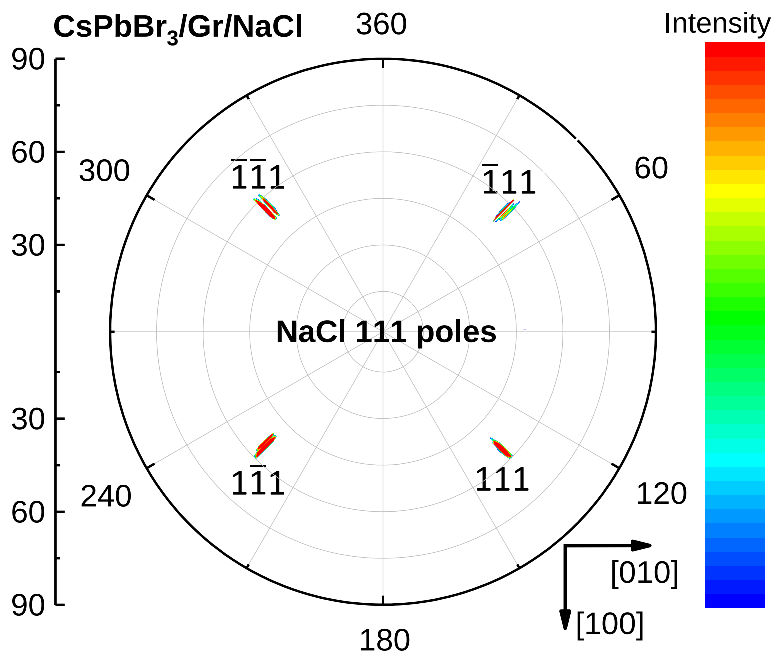

Supplementary Figure 4 X-ray pole figure of NaCl 111 from $CsPbBr_3$/Gr/NaCl.

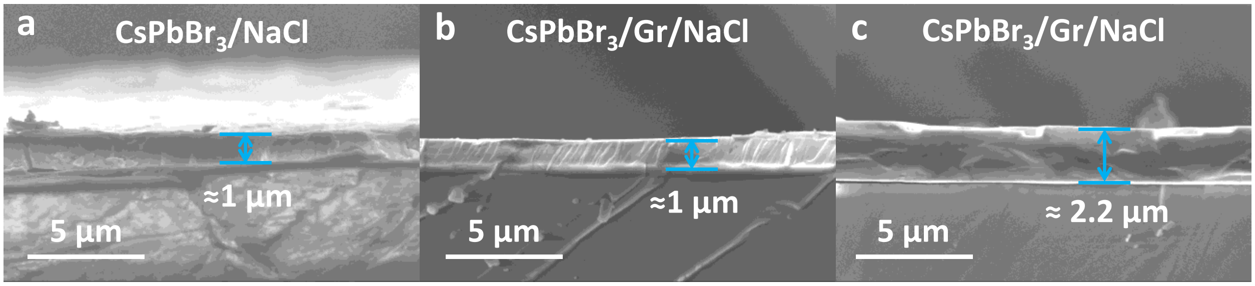

Supplementary Figure 5 Cross-sectional SEM images of $CsPbBr_3$/NaCl (**a**) and $CsPbBr_3$/Gr/NaCl (**b** and **c**) thin films.

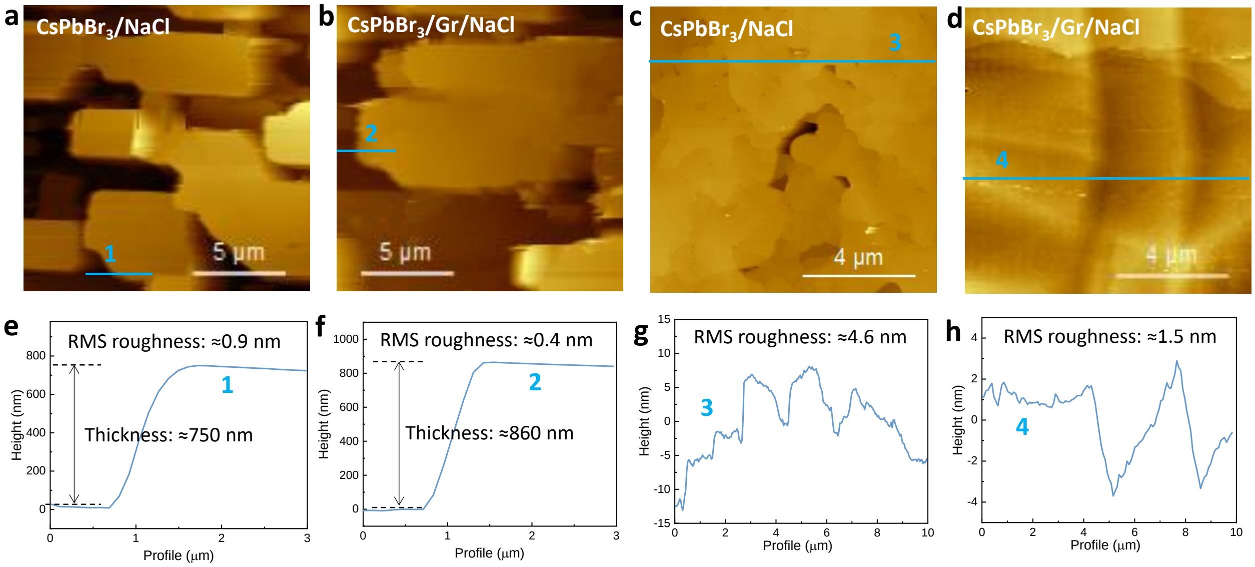

Supplementary Figure 6 AFM images of flakes in $CsPbBr_3$/NaCl (**a**) and $CsPbBr_3$/Gr/NaCl (**b**), and thin films in $CsPbBr_3$/NaCl (**c**) and $CsPbBr_3$/Gr/NaCl (**d**). The few nanometers step heights in (c) and (d) may be due to different domains merging together.

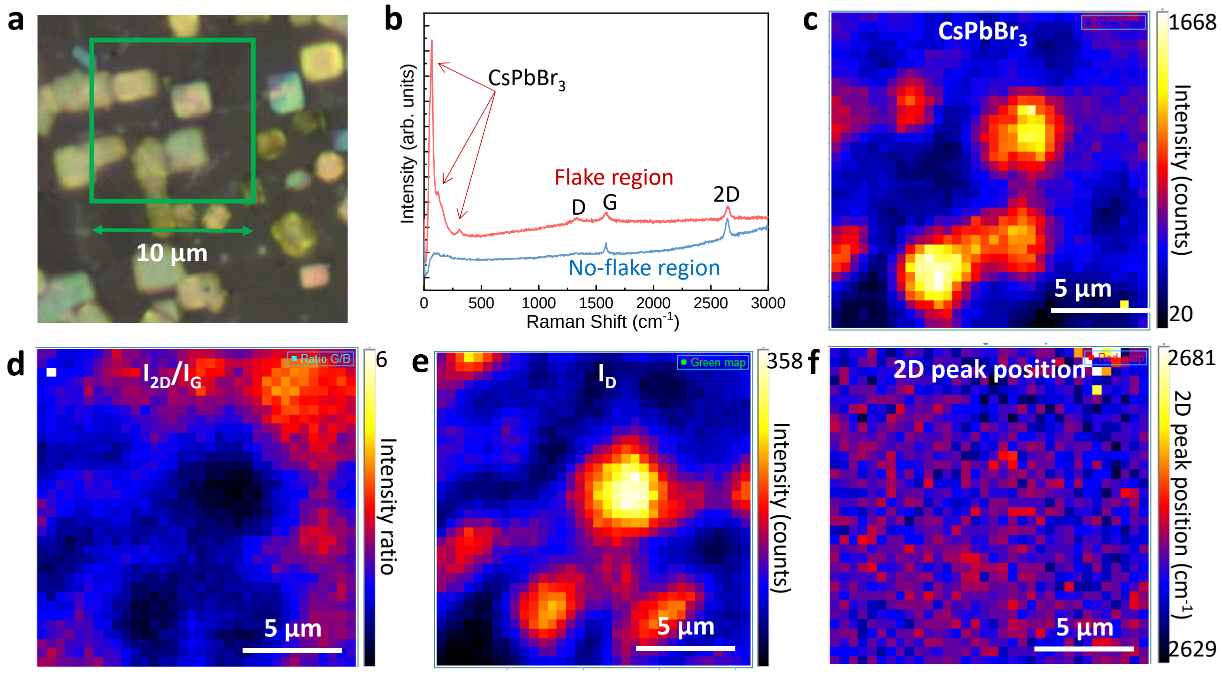

Supplementary Figure 7 Raman measurements on $CsPbBr_3$/Gr/NaCl. **a-c**, Optical image (**a**), Raman spectrum (**b**), Raman mapping (**c**) of remote epitaxial $CsPbBr_3$ flakes. The mapping area (10×10 µm^2) is indicated in green square in **a**. **d-f**, Raman mapping of I_{2D}/I_G (**d**), I_D (**e**) and 2D peak position (**f**) of graphene.

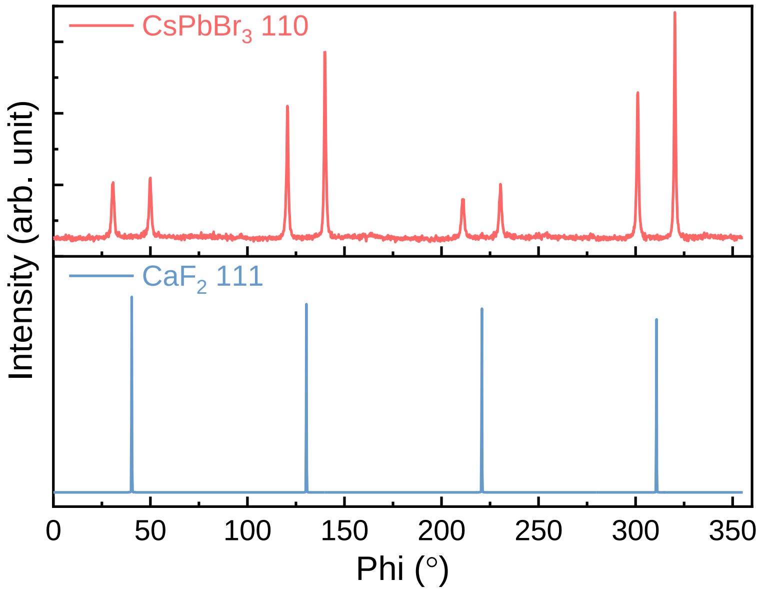

Supplementary Figure 8 φ-scan of $CsPbBr_3$ 110 and CaF_2 111 from $CsPbBr_3$/Gr/CaF_2.

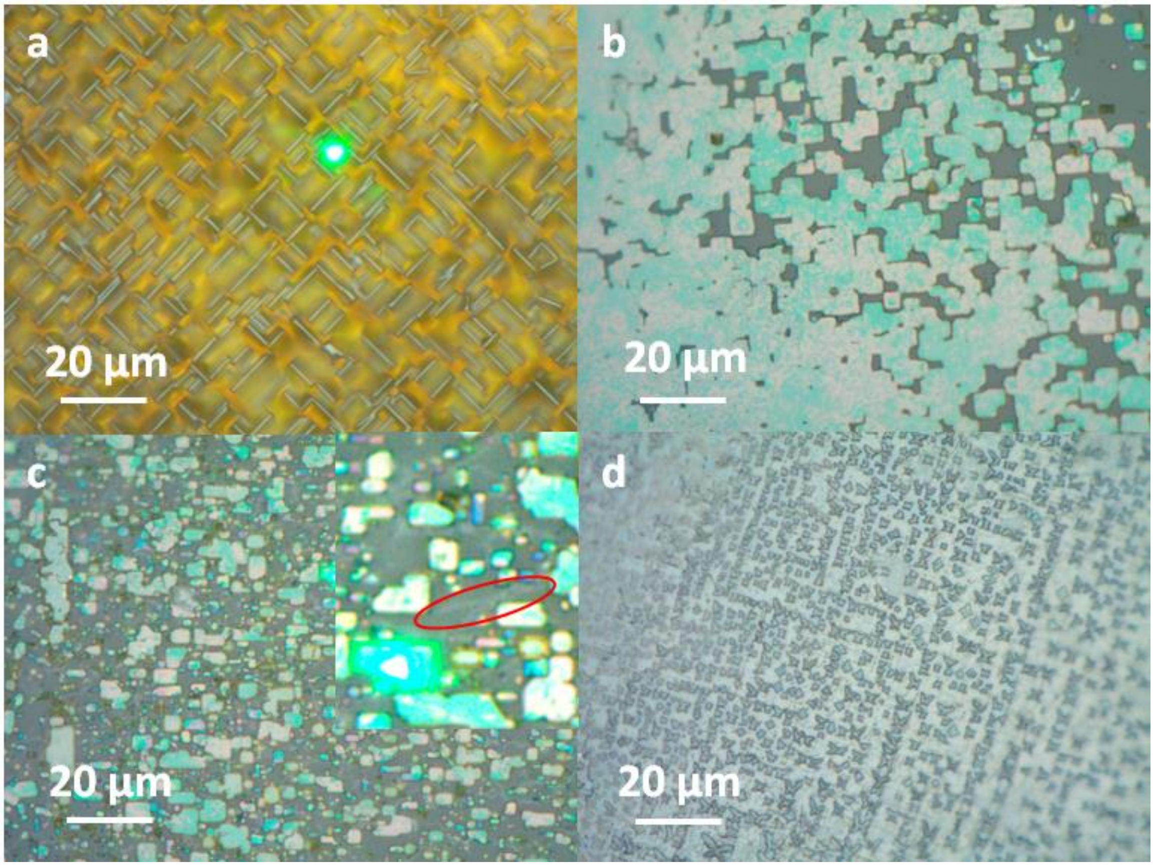

Supplementary Figure 9 Optical images of $CsPbBr_3$/Gr/CaF_2 (**a**), $CsPbBr_3$/NaCl (**b**), $CsPbBr_3$/Gr/NaCl (**c**), NaCl after etching (**d**). Inset of (c) shows a typical finding that nucleation often does not occur at the wrinkle of graphene. At the wrinkles of graphene, the substrate-film coupling strength could be weaker due to the larger film-substrate distance compared to non-defective region since electrostatic interaction (both van der Waals and ionic) decays as distance increases. In our case, the wrinkles of graphene can still be seen among the remote epitaxial $CsPbBr_3$ flakes after growth, as indicated in red ellipses in the optical microscopy image of (c). Our experimental observation indicates that the activation energy for nucleation at graphene/substrate surface seems to be even lower than that at wrinkles. Hence, in the remote epitaxy in our case, the growth kinetics might be dominated by the polar substrates while the wrinkles of graphene play a minor role.

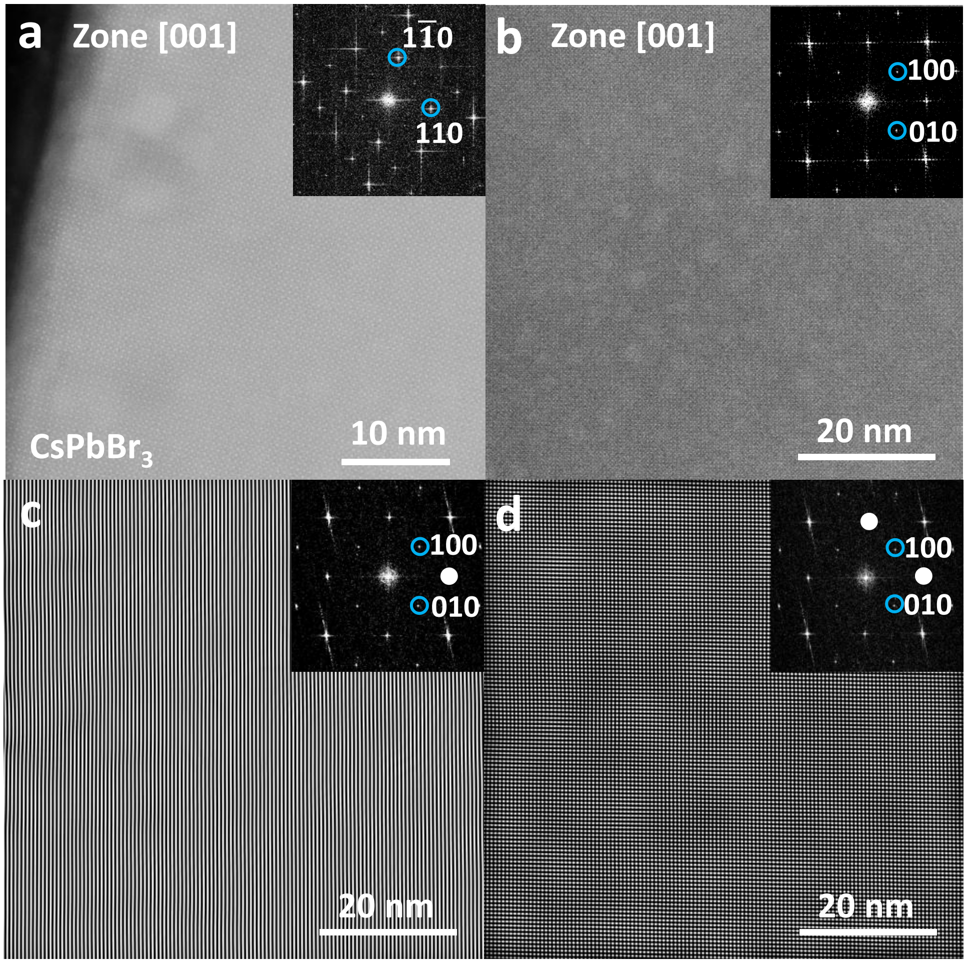

Supplementary Figure 10 STEM images for $CsPbBr_3$/Gr/NaCl at different regions with FFT in insets (**a-b**) and inverse FFTs (**c** and **d**) of their insets. The insets in **c** and **d** are FFTs of **b** with additional white spots.

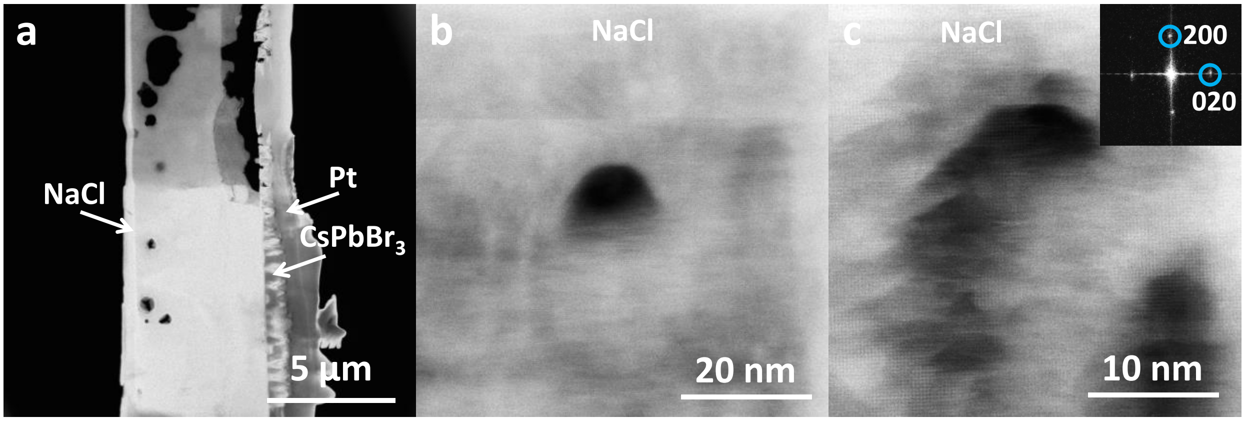

Supplementary Figure 11 STEM images of $CsPbBr_3$/Gr/NaCl (**a**) and NaCl (**b** and **c**).

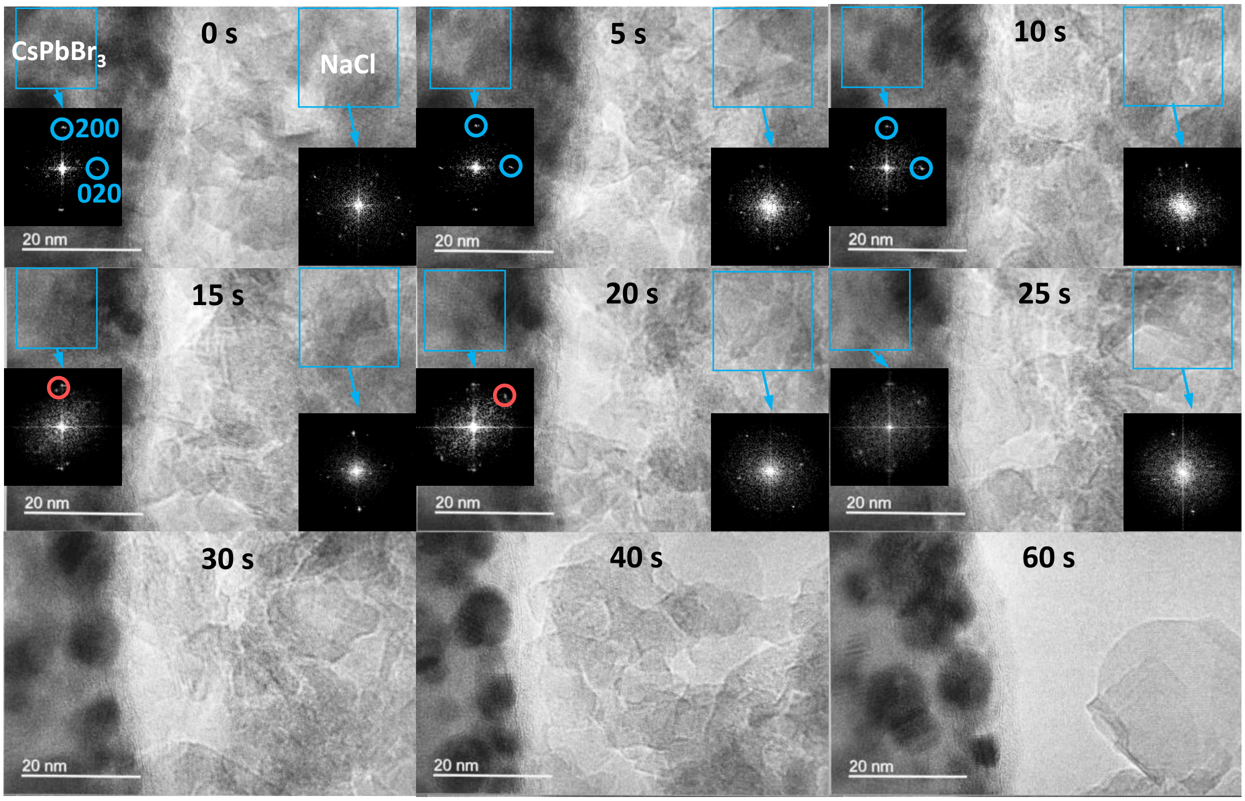

Supplementary Figure 12 TEM images of $CsPbBr_3$/Gr/NaCl and FFTs in their insets with increasing time.

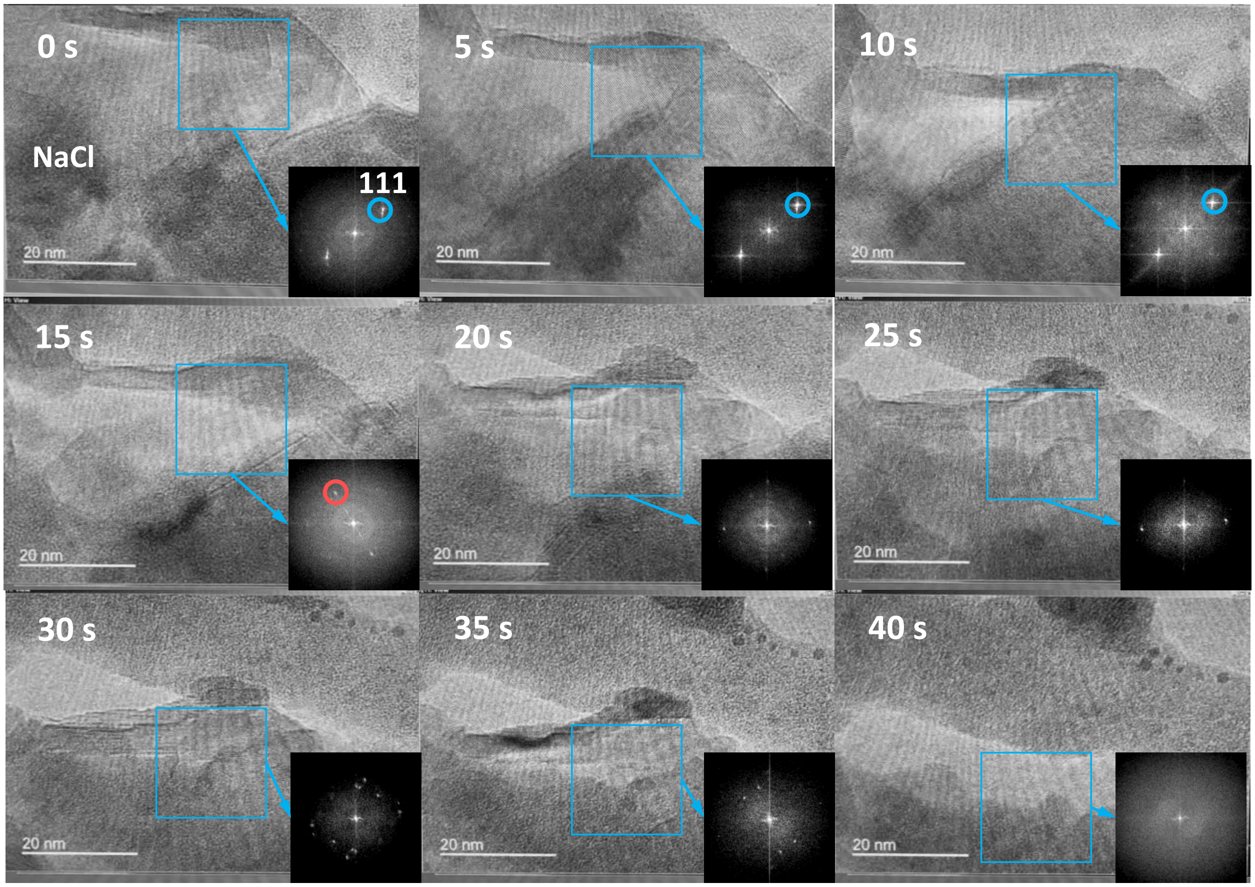

Supplementary Figure 13 TEM images of NaCl and FFTs in their insets with increasing time.

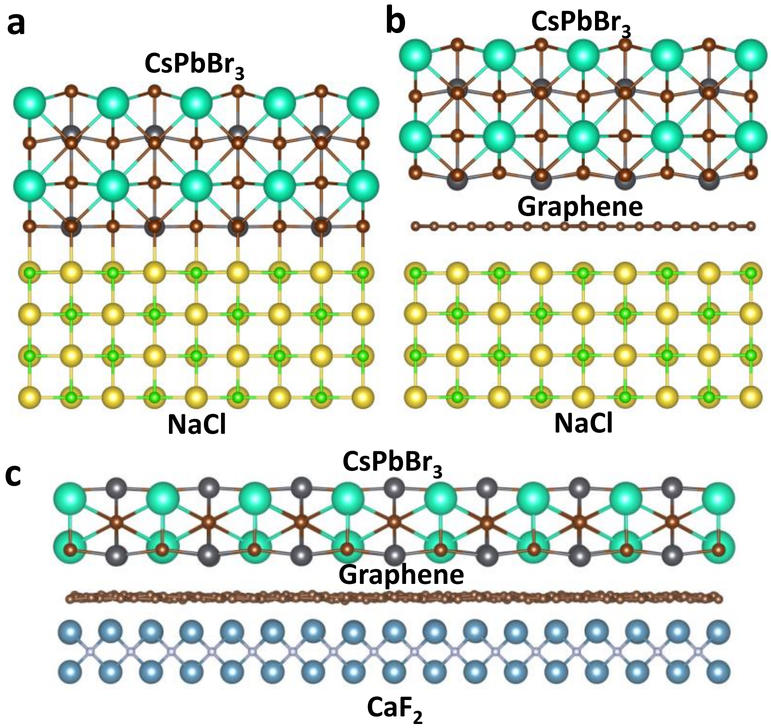

Supplementary Figure 14 DFT simulation models of $CsPbBr_3$(001)/NaCl(001) (**a**) $CsPbBr_3$(001)/Gr/NaCl(001) (**b**) and $CsPbBr_3$(011)/Gr/CaF_2(001) (**c**).

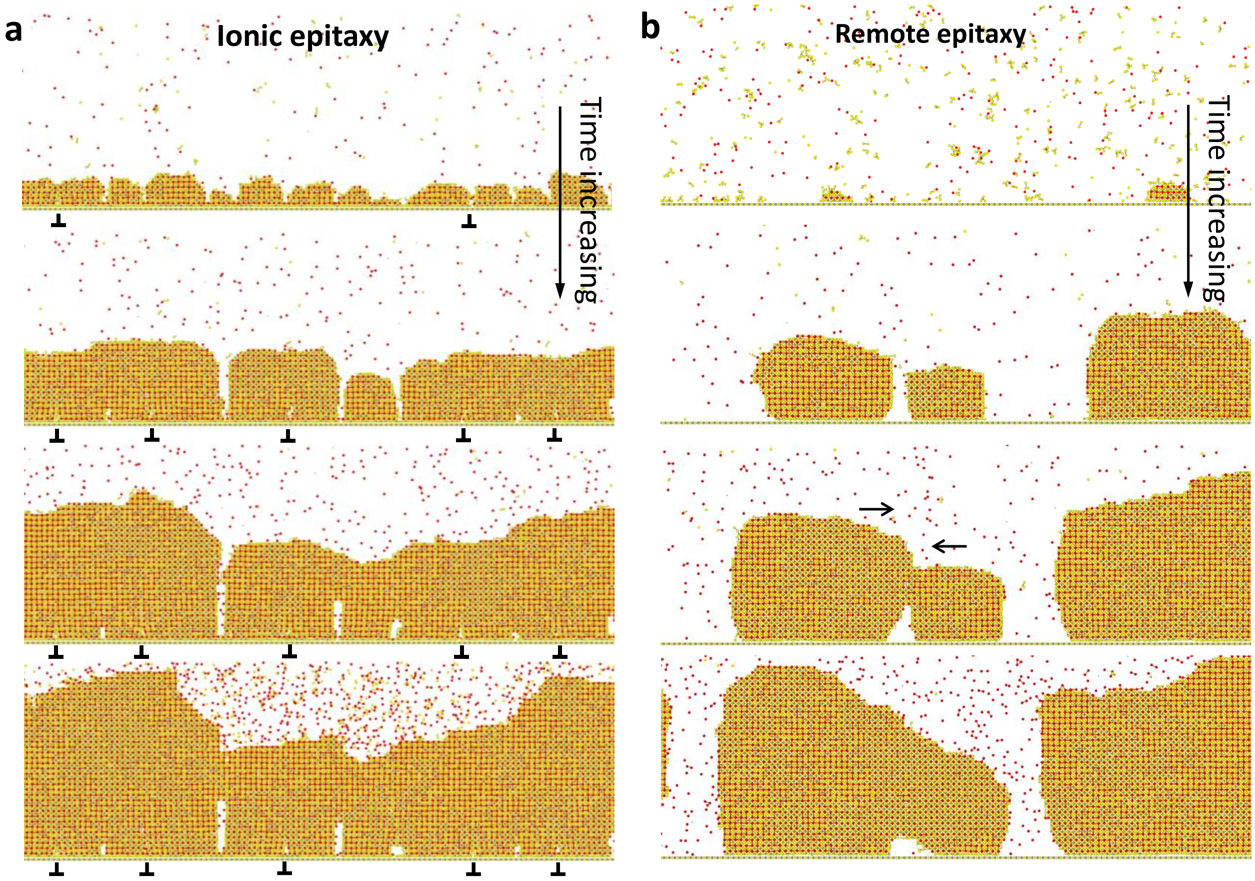

Supplementary Figure 15 Side views of MD simulations for ionic epitaxy (**a**) and remote epitaxy (**b**).

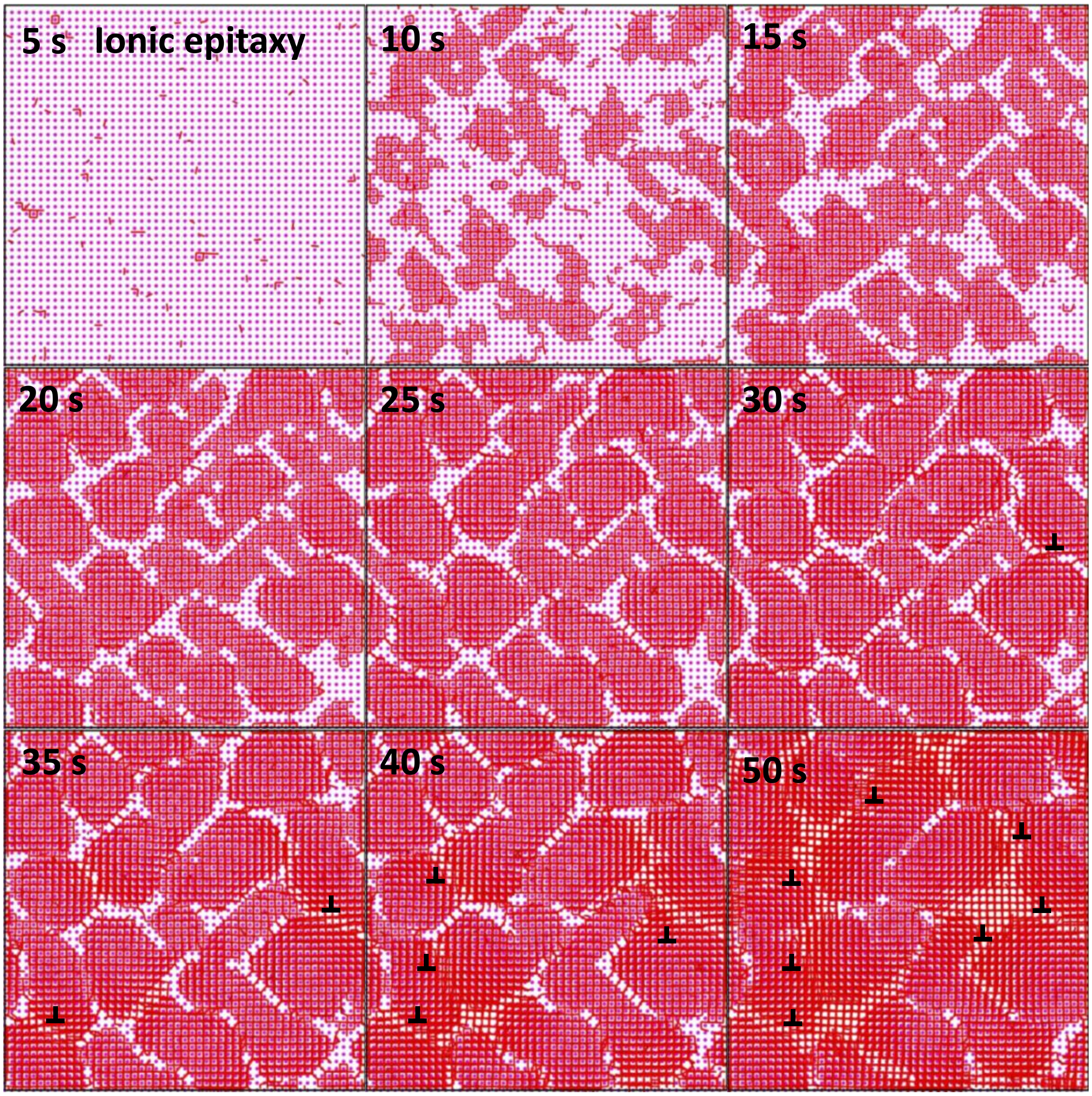

Supplementary Figure 16 Top view of MD simulations for ionic epitaxy.

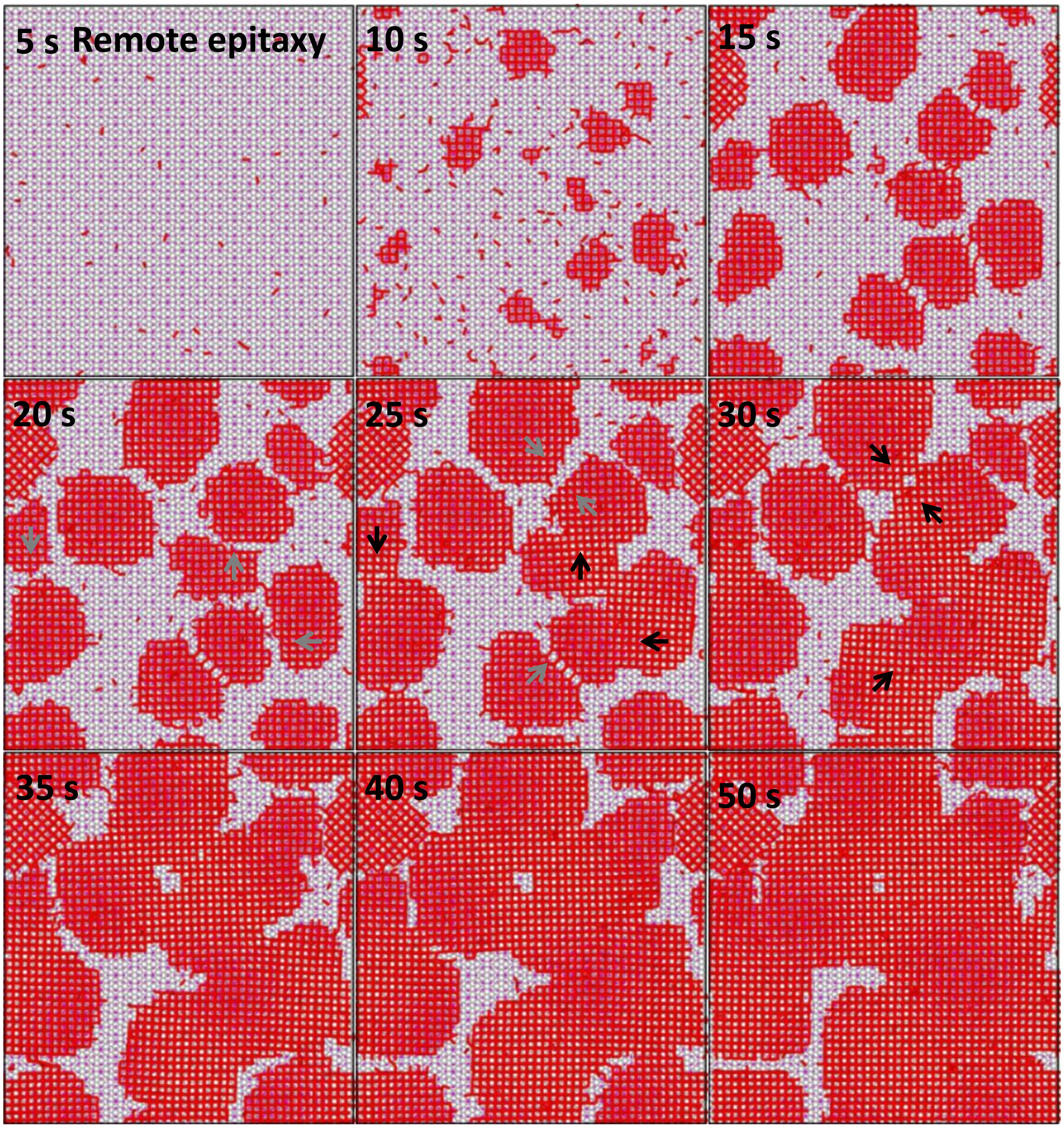

Supplementary Figure 17 Top view of MD simulations for remote epitaxy.

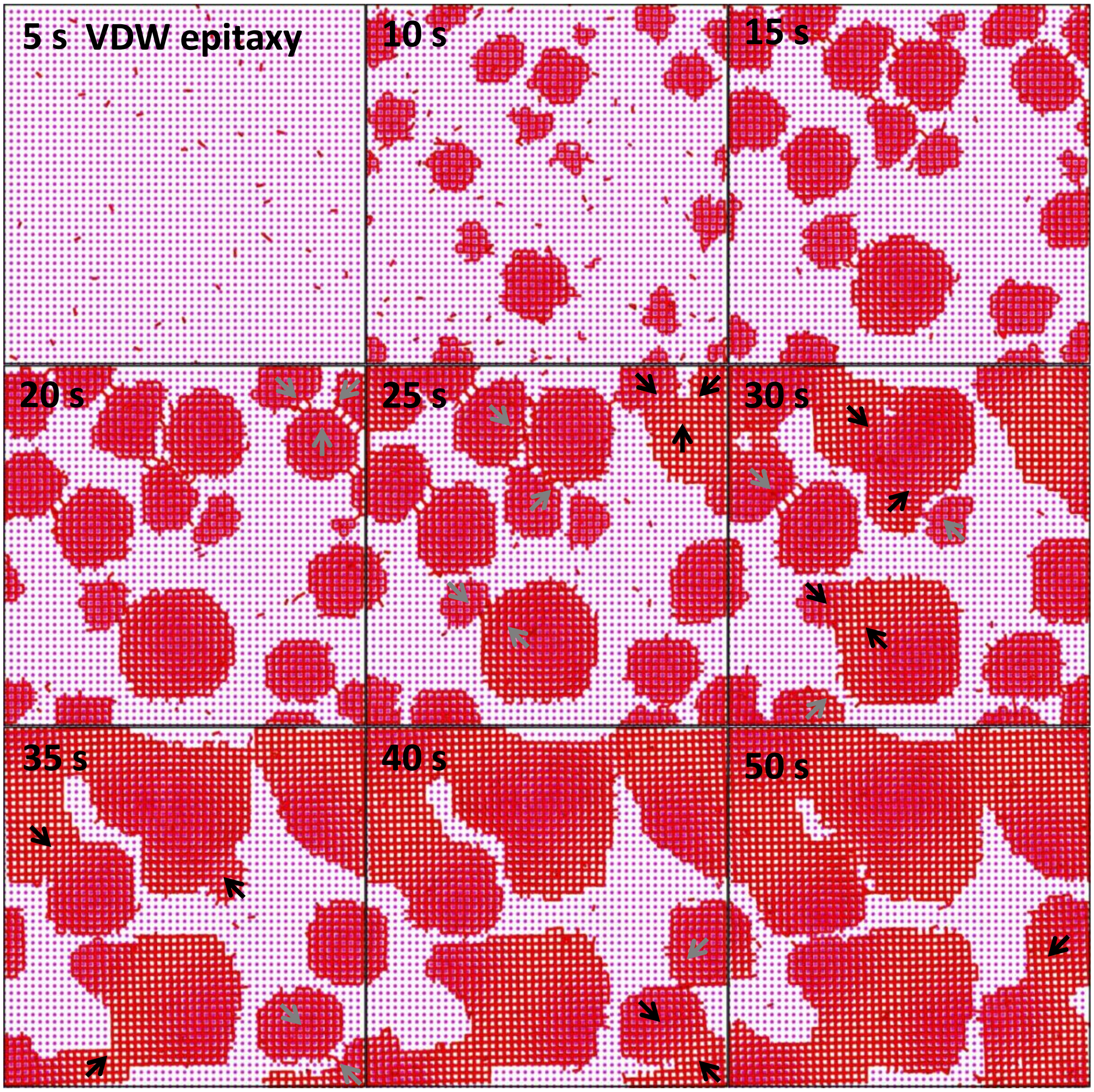

Supplementary Figure 18 Top view of MD simulations for van der Waals (VDW) epitaxy.

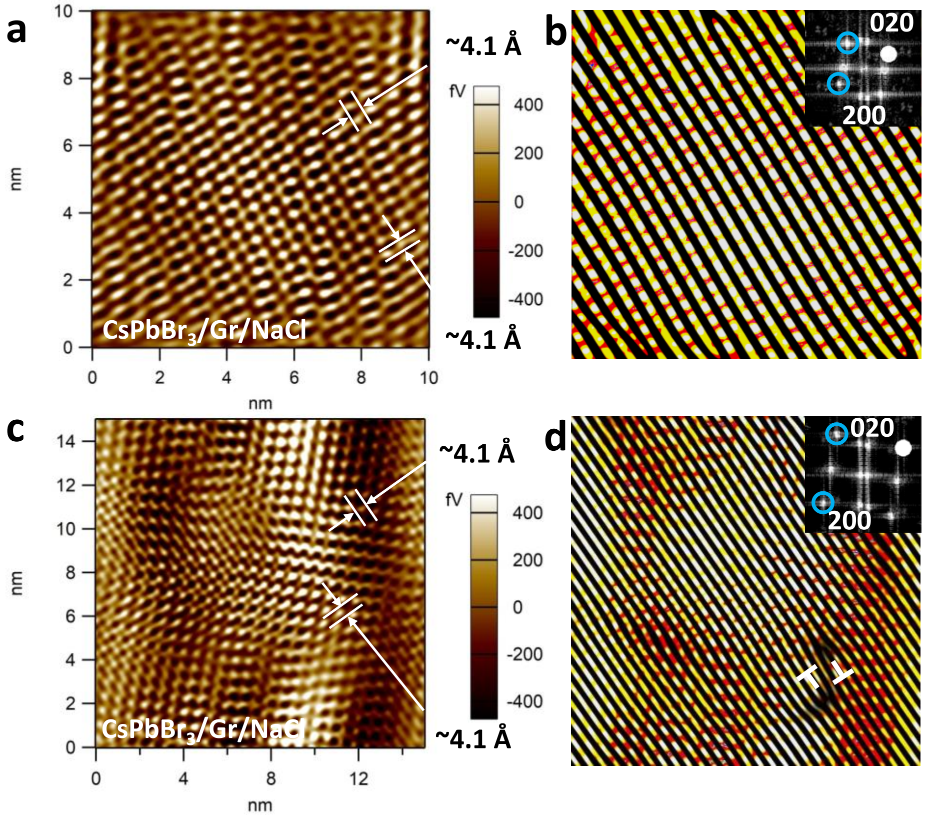

Supplementary Figure 19 HRAFM images for $CsPbBr_3$/Gr/NaCl at different surface regions (**a** and **c**) and inverse FFTs (**b** and **d**) of their insets. The insets in **b** and **d** are FFTs of **a** and **c** with additional white spots. Dislocations are indicated in **d**.

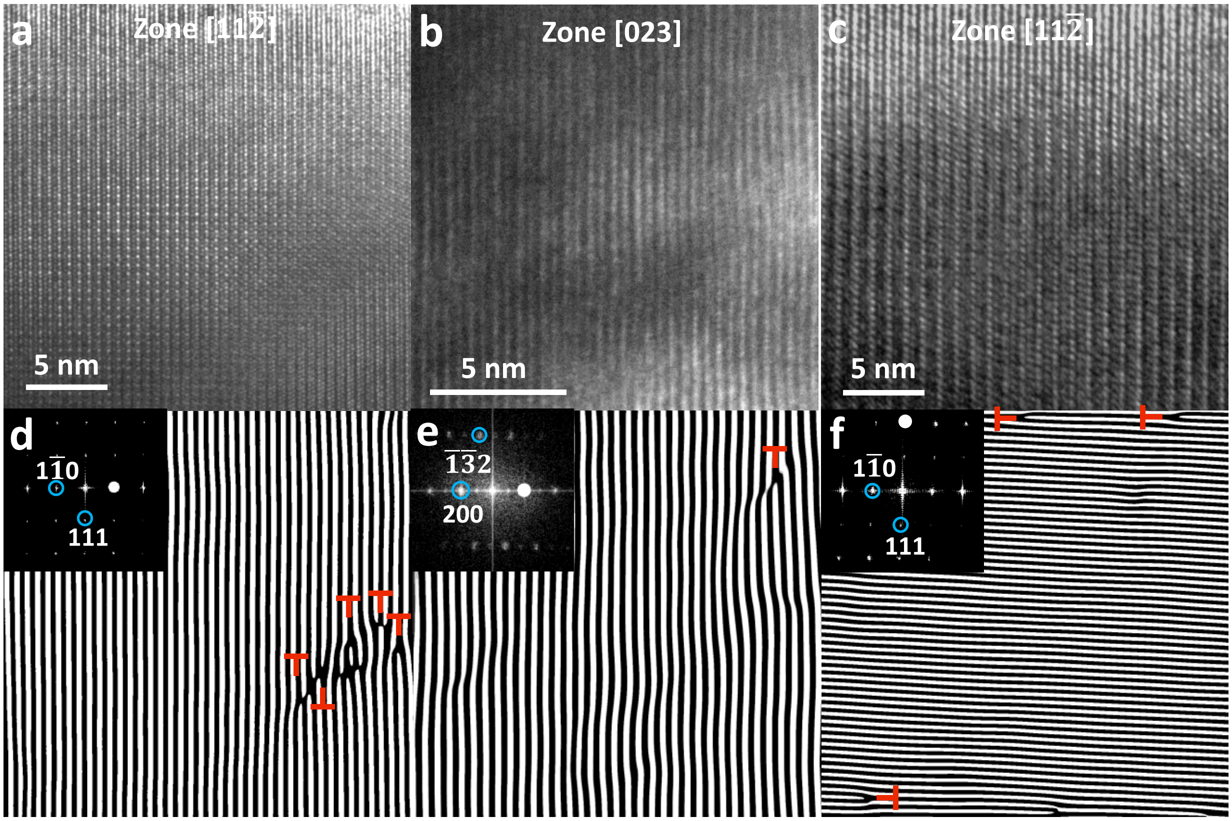

Supplementary Figure 20 HRTEM images for $CsPbBr_3$/Gr/NaCl at different regions (**a-c**) and inverse FFTs (**d-f**) of their insets. The insets in **d-f** are FFTs from **a-c** with additional white spots, respectively. Dislocations are indicated in **d-f**.

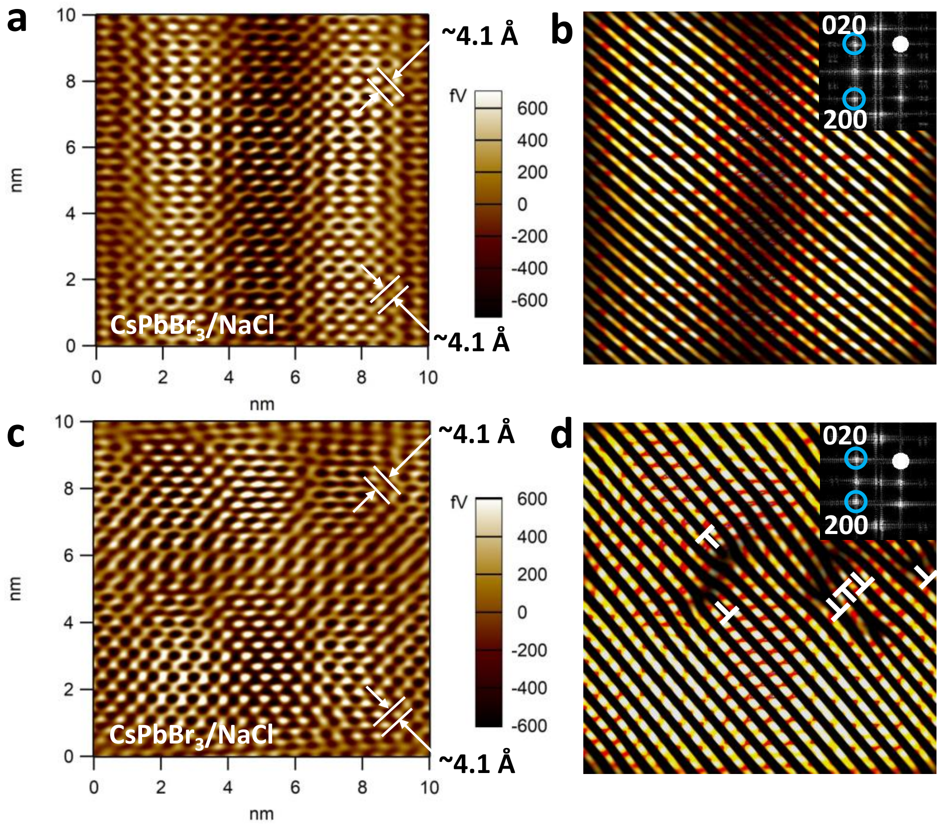

Supplementary Figure 21 HRAFM images for $CsPbBr_3$/NaCl at different surface regions (**a** and **c**) and inverse FFTs (**b** and **d**) of their insets. The insets in **b** and **d** are FFTs of **a** and **c** with additional white spots. Dislocations are indicated in **d**.

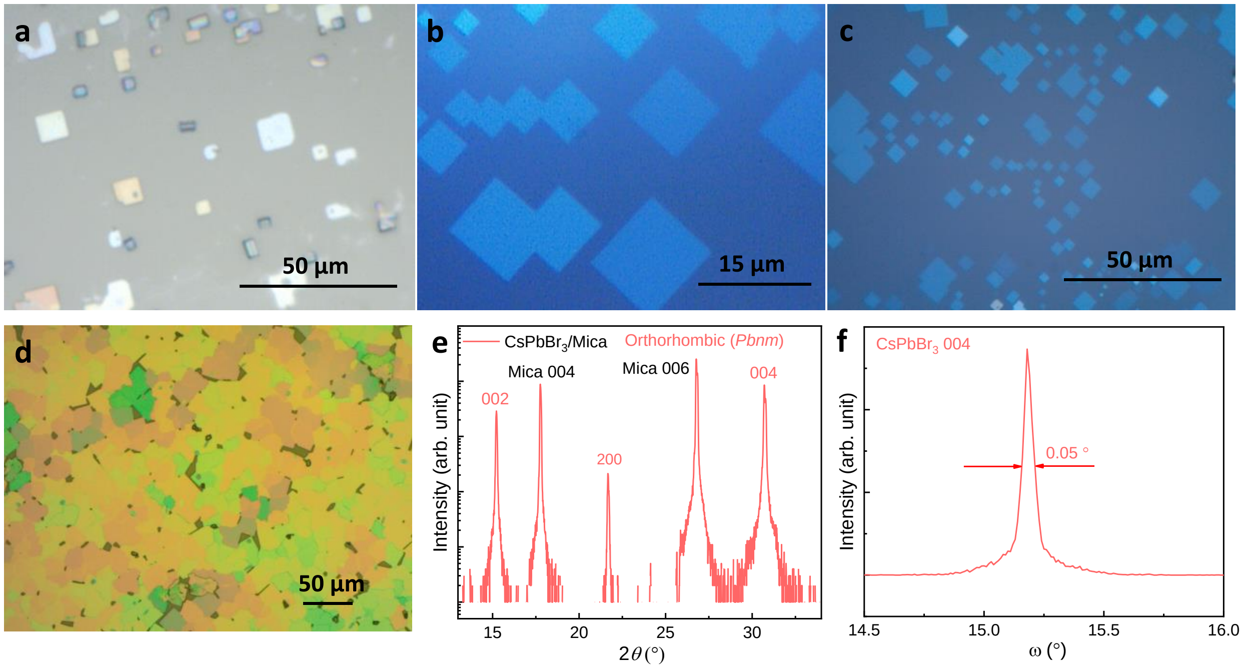

Supplementary Figure 22 vdW epitaxy of halide perovskite. Optical microscopy images of halide perovskite flakes grown on Gr/Si(100) (a) and mica (b-d), and XRD θ-2θ scanning of $CsPbBr_3$/Mica (e) and rocking curve of $CsPbBr_3$ 004 (f).

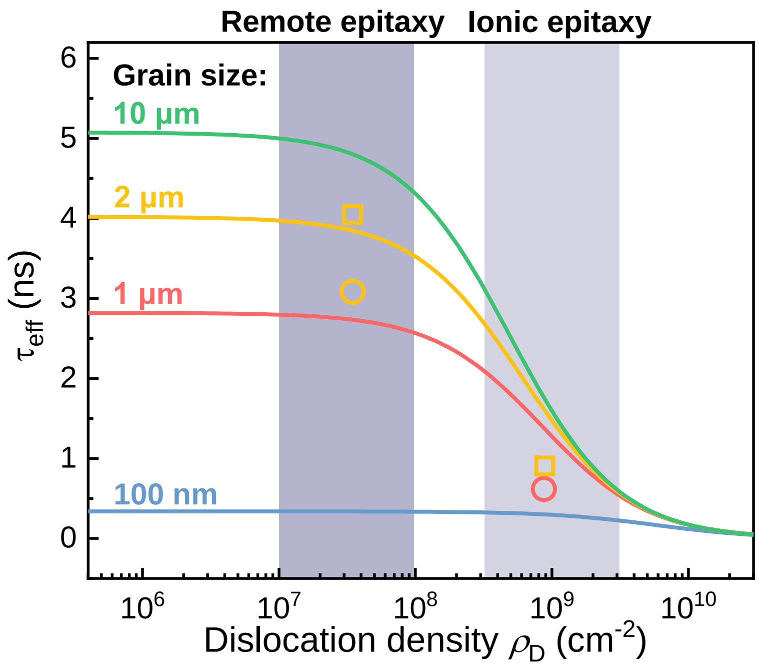

Supplementary Figure 23 Effective carrier lifetime as a function of dislocation density with different grain size. Experimental data for thin film and flake are indicated in circle and square, respectively. Remote epitaxy and ionic epitaxy regions are painted in dark purple and light purple, respectively.

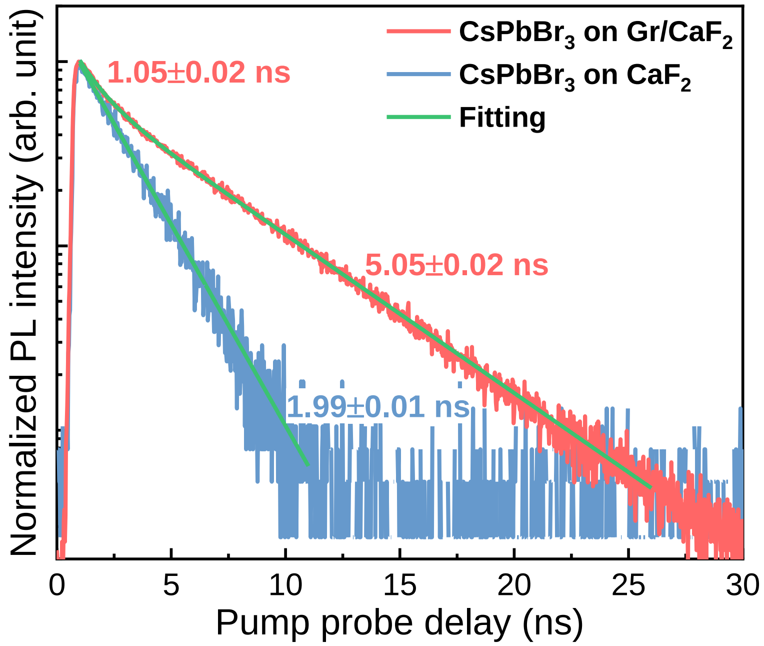

Supplementary Figure 24 TRPL of $CsPbBr_3$ on CaF_2 in both remote and ionic epitaxy.

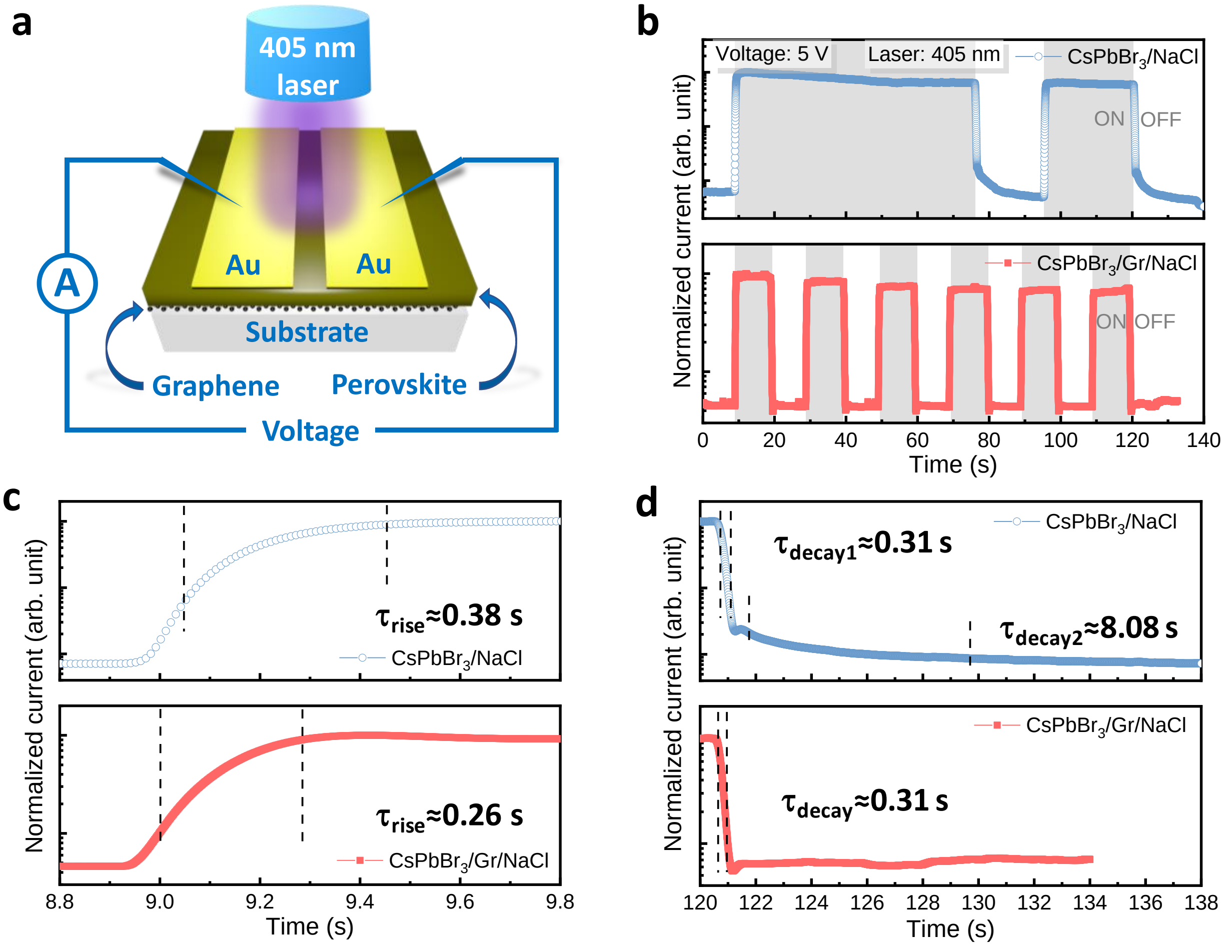

Supplementary Figure 25 **a**, A photodector (Au/$CsPbBr_3$/Au) made by gold contacts on top of thin film. **b**, Photo response on both devices made by ionic and remote epitaxial thin film. **c**, **d**, Enlarged rising (**c**) and falling (**d**) parts of current for both devices. $CsPbBr_3$/NaCl and $CsPbBr_3$/Gr/NaCl show different decay trends: an additional decay tail with a long decay time of around 8.08 s can be observed in the ionic epitaxial thin film-based device.

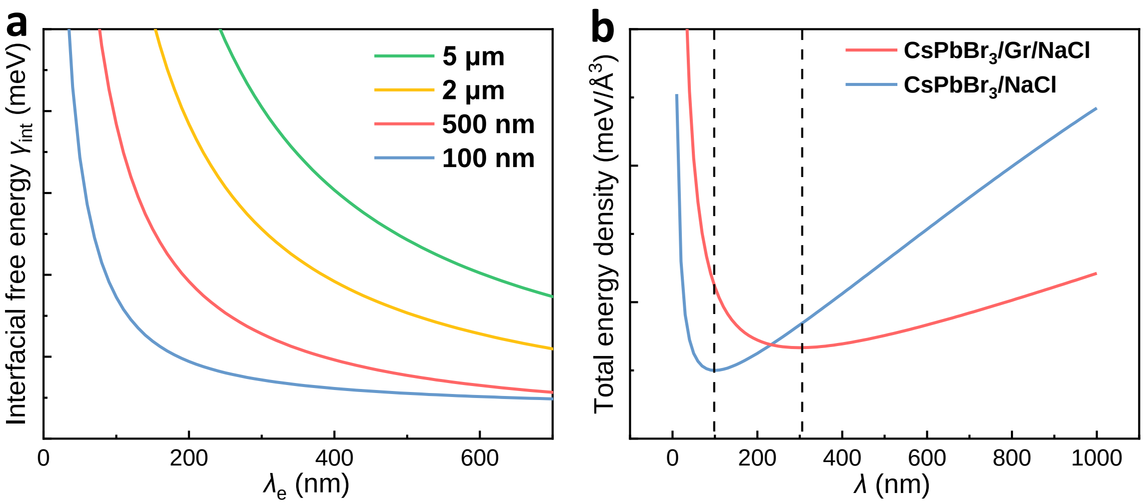

Supplementary Figure 26 **a**, Interfacial free energy as a function of spatial periodicity of the ferroelastic domain. **b**, Total energy density as a function of λ in both ionic and remote epitaxy.

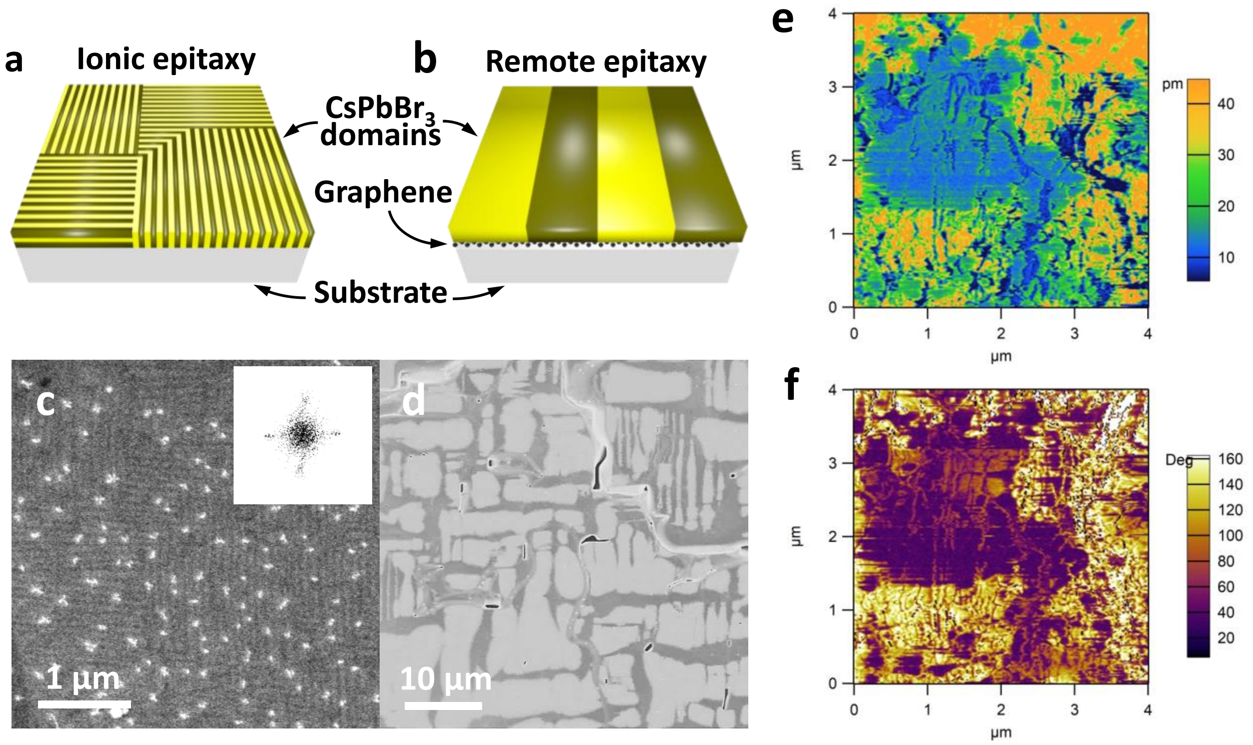

Supplementary Figure 27 Ferroelastic domain patterns in ionic and remote epitaxy. **a**,**b**, Schematic illustrations of ferroelastic domain patterns in ionic epitaxy (**a**) and remote epitaxy (**b**), respectively. **c**,**d**, SEM images of the striped ferroelastic domains in ionic epitaxy (**c**) and remote epitaxy (**d**), respectively. **e**,**f**, PFM amplitude (**e**) and phase (**f**) mapping of the piezoresponse for a local area on the remote epitaxial $CsPbBr_3$ thin film.

Supplementary Tables

Supplementary Table 1 Pair-dependent LJ potential parameters for ABC_3 perovskite system.

	Interaction Type	$\varepsilon_{\alpha\beta}/\varepsilon_{BC}$	$\sigma_{\alpha\beta}/\sigma_{BC}$	$r_{\alpha\beta,c}/\sigma_{BC}$
A-A	Repulsive only	1.0	1.89553	2.12766
A-B	Repulsive only	1.0	1.69600	1.90370
A-C	With attraction	0.4	1.41321	2.01568
B-B	Repulsive only	1.0	2.19482	2.46361
B-C	With attraction	1.0	1.00000	2.01568
C-C	Repulsive only	1.0	1.34682	1.51176
C-D (Substrate I)	With attraction	2.0	1.67973	2.12766
C-D (Substrate II)	With attraction	2.0	1.67973	2.12766
A-F, B-F, D-F, E-F (Substrate II)	Repulsion only	0.1	0.79812	0.89586
C-F (Substrate II)	With attraction	0.1	0.89586	1.67973
C-D (Substrate III)	With attraction	1.0	1.67973	2.12766
All other interactions	Repulsive only	1.0	1.34682	1.51176

Supplementary Notes

Supplementary Note 1

Controlling domain wavelength via remote epitaxy

It is in debate that the ferroelasticity or ferroelectricity in hybrid organic-inorganic perovskites plays a potential role to underpin the desirable device performance[1, 2, 3, 4, 5]. The striped ferroelastic domains in methylammonium lead iodide perovskite have been recently observed explained as a result of the cubic-tetragonal phase transition[1, 3, 6]. A similar ferroelastic domain structure is naturally expected in inorganic halide perovskites like $CsPbBr_3$ as a result of the cubic-tetragonal-orthorhombic phase transition. Controlling the dimension and wavelength of the domain structures may serve as a solution to engineer the device performance. In this study, it is observed that ionic epitaxy and remote epitaxy lead to completely different dimensions and wavelength.

To explain our observation, we have applied continuum mechanics modeling. With the assumption that both domains coexist in equal fraction, the total energy density E is given by[7]:

$$E(\lambda) = \frac{\lambda\epsilon}{\pi^3}\sum_{j=0}^{\infty}\frac{1-e^{-2(2j+1)\pi t/\lambda}}{(2j+1)^3} + \frac{\gamma t}{\lambda} + f_{\mathrm{t}}t, \qquad (1)$$

where λ is the spatial period of the domain pattern, t is thickness of the thin film, and f_{t} is the free energy densities of the tetragonal phase, respectively. γ is the domain wall energy density. ϵ is the volume density of the elastic strain energy, which is dependent on substrate-film coupling strength. Thus, to compare the elastic strain energy density in both ionic and remote epitaxy, since much smaller contribution from the two domains themselves due to the close lattice constants of a and c, we introduced a simplified system with only one domain grown on substrate. Epitaxial growth changes the total energy density of the simplified system ΔE_{α}, which is increased by the elastic strain energy density and released by bonding energy density. Assuming dangling bonds would be formed if a strain less than lattice misfit is applied, the ratio of bonding energy density to interfacial free energy density can be estimated to be the bonding atoms' percentage on the substrate, which is $a_{\mathrm{f}}(1+\alpha)/a_{\mathrm{s}}$. Assuming that strain is uniformly distributed in the film, ΔE_{α} is given by:

$$\Delta E_{\alpha} = -\frac{a_{\mathrm{f}}(1+\alpha)}{a_{\mathrm{s}}}\frac{\gamma_{\mathrm{int}}}{t} + \epsilon \approx -\frac{a_{\mathrm{f}}(1+\alpha)}{a_{\mathrm{s}}}\frac{\gamma_{\mathrm{int}}}{t} + \frac{1}{2}Y\alpha^2, \qquad (2)$$

where α is the strain in film, γ_{int} is the interfacial free energy taken from DFT calculation in Fig. 2c and also mentioned in Eq. (1) in main text, and Y is the Young's modulus of the $CsPbBr_3$ (16 GPa[8]). By minimizing ΔE_{α}, the strain α is given by:

$$\alpha = \frac{a_{\mathrm{f}}\gamma_{\mathrm{int}}}{2a_{\mathrm{s}}Yt}. \qquad (3)$$

Hence, the volume density of the elastic strain energy ϵ is proportional to γ_{int}^2. After determining the equilibrium domain period λ_{e} by minimizing $E(\lambda)$, the interfacial free energy γ_{int} can be plotted as a function of λ_{e} with different sample thicknesses, as shown Supplementary Figure 26a, indicating the increase of domain period with decreasing interfacial free energy and larger domain periods in thick films.

Therefore, the remote epitaxy theoretically would exhibit larger domain period due to the weaker interfacial energy, compering to the ionic epitaxy.

Schematic illustrations of domain patterns are shown in Supplementary Figure 27a and b for ionic and remote epitaxy, respectively. To the best of our knowledge, there is no experimental observation on the ferroelastic domain pattern in the inorganic halide perovskite $CsPbBr_3$. Here, in accordance with theoretical modeling, we have observed striped ferroelastic domains in $CsPbBr_3$ from both ionic and remote epitaxy (with different thicknesses), as shown in Supplementary Figure 27c and d. A FFT pattern was shown in the inset of Supplementary Figure 27c, giving the spatial period of the domain patterns of around 95 nm. For the remote epitaxy in Supplementary Figure 27d, the ferroelastic domain pattern was greatly changed and the domain period was increased to between 1 and 5 μm. For both ionic and remote epitaxy, the total energy densities have been plotted as a function of λ, as shown in Supplementary Figure 26b, giving consistent trend with the experimental observation. The ferroelastic domains can be characterized by PFM measurement[6]. Supplementary Figure 27e and f show the PFM amplitude and phase mapping of the piezoresponse for a local area on the remote epitaxial thin film, respectively, confirming ferroelastic domains in $CsPbBr_3$.

Supplementary References

1. Liu Y, *et al.* Chemical nature of ferroelastic twin domains in $CH_3NH_3PbI_3$ perovskite. *Nat. Mater.* **17**, 1013-1019 (2018).
2. Röhm H, Leonhard T, Hoffmann MJ, Colsmann A. Ferroelectric domains in methylammonium lead iodide perovskite thin-films. *Energy Environ. Sci.* **10**, 950-955 (2017).
3. Strelcov E, *et al.* $CH_3NH_3PbI_3$ perovskites: Ferroelasticity revealed. *Sci. Adv.* **3**, e1602165 (2017).
4. Kutes Y, Ye L, Zhou Y, Pang S, Huey BD, Padture NP. Direct Observation of Ferroelectric Domains in Solution-Processed $CH_3NH_3PbI_3$ Perovskite Thin Films. *J. Phys. Chem. Lett.* **5**, 3335-3339 (2014).
5. Huang B, *et al.* Ferroic domains regulate photocurrent in single-crystalline $CH_3NH_3PbI_3$ films self-grown on FTO/TiO_2 substrate. *npj Quantum Materials* **3**, 30 (2018).
6. Hermes IM, *et al.* Ferroelastic Fingerprints in Methylammonium Lead Iodide Perovskite. *The Journal of Physical Chemistry C* **120**, 5724-5731 (2016).
7. Wu J, Gu Q, Guiton BS, de Leon NP, Lian O, Park H. Strain-induced self organization of metal-insulator domains in single-crystalline VO2 nanobeams. *Nano Lett.* **6**, 2313-2317 (2006).
8. Rakita Y, Cohen SR, Kedem NK, Hodes G, Cahen D. Mechanical properties of APbX3 (A = Cs or CH_3NH_3; X = I or Br) perovskite single crystals. *MRS Communications* **5**, 623-629 (2015).
