# Supplementary material for: Carrier lifetime enhancement in halide perovskite via remote epitaxy
Source: Nat Commun. 2019 Sep 12;10:4145. doi: 10.1038/s41467-019-12056-1 (PMC6742762; doi:10.1038/s41467-019-12056-1)
Supplement: Supplementary file 3 — Description of Additional Supplementary Files [file 41467_2019_12056_MOESM3_ESM.pdf]

# Description of Additonal Supplementary Files

Supplementary Movie 1: HRTEM $CsPbBr_3$-Gr-NaCl

Supplementary Movie 2: HRTEM NaCl

Supplementary Movie 3: HRTEM NaCl diffraction pattern

Supplementary Movie 4: Side view of ionic epitaxy by molecular dynamics simulation

Supplementary Movie 5: Side view of remote epitaxy by molecular dynamics simulation

Supplementary Movie 6: Side view of van der Waals epitaxy by molecular dynamics simulation

Supplementary Movie 7: Top view of ionic epitaxy by molecular dynamics simulation

Supplementary Movie 8: Top view of remote epitaxy by molecular dynamics simulation

Supplementary Movie 9: Top view of van der Waals epitaxy by molecular dynamics simulation
